# Preliminary Experience in Ultra-High Frequency Ultrasound Assessment of Cutaneous Primary Lymphomas: An Innovative Classification

**DOI:** 10.3390/cancers16132456

**Published:** 2024-07-04

**Authors:** Anna Russo, Vittorio Patanè, Federico Gagliardi, Fabrizio Urraro, Andrea Ronchi, Paola Vitiello, Antonello Sica, Giuseppe Argenziano, Valerio Nardone, Alfonso Reginelli

**Affiliations:** 1 Department of Precision Medicine, University of Campania Luigi Vanvitelli, 80131 Naples, Italy; 2 Pathology Unit, Department of Mental and Physical Health and Preventive Medicine, Università degli Studi della Campania “Luigi Vanvitelli”, 80138 Naples, Italy; 3 Dermatology Unit, Department of Mental and Physical Health and Preventive Medicine, University of Campania Luigi Vanvitelli, 80131 Naples, Italy; paola.vitiello@unicampania.it (P.V.);

**Keywords:** high-frequency ultrasound, ultra-high frequency ultrasound, primary cutaneous lymphoma, oncologic ultrasonography

## Abstract

**Simple Summary:**

Primary cutaneous lymphomas (PCLs) are rare forms of skin cancer arising from certain types of white blood cell. They affect the skin without spreading to other parts of the body initially. Understanding them is crucial as they require different treatments compared to other skin cancers. However, diagnosing PCLs is tricky because they can look similar to other skin conditions. We are proposing to use a type of ultrasound called high-frequency ultrasound (HFUS) to better understand how PCLs appear under the skin. By doing this, we hope to improve our ability to identify PCLs accurately and quickly. This could lead to better treatment decisions and outcomes for patients with PCLs.

**Abstract:**

Background: Primary cutaneous lymphoma (PCL) is a rare form of extranodal non-Hodgkin’s lymphoma characterized by malignant lymphocytes confined to the skin. Accurate diagnosis and staging are crucial for optimal management, yet radiological literature on imaging PCL remains limited. This study aims to delineate the imaging characteristics of PCLs using high and ultra-high frequency ultrasound (UHFUS) and proposes a classification system based on ultrasound findings. Methods: A cohort of 88 individuals with suspected PCL underwent high-resolution ultrasound (HRUS) and color Doppler examination of lesions. Lesions were categorized based on sonographic appearance, and subsequent histopathological assessment confirmed the diagnosis. Results: Ultrasound imaging revealed distinct patterns for primary cutaneous T-cell lymphomas (PCTCL) and primary cutaneous B-cell lymphomas (PCBCL), with characteristic features such as hypoechoic nodules, pseudonodular lesions, and dermal infiltration. Histopathological analysis confirmed the ultrasound findings, supporting the proposed classification system. Conclusions: Ultrasonography, particularly UHFUS, offers valuable insights into the imaging characteristics of primary cutaneous lymphomas, aiding the accurate diagnosis and assessment of treatment response. The proposed classification system based on ultrasound findings enhances the diagnostic approach to PCLs, and paves the way for improved patient care and management strategies.

## 1. Introduction

Primary cutaneous lymphoma (PCL) is a rare form of extranodal non-Hodgkin’s lymphoma (NHL) characterized by malignant lymphocytes confined to the skin without the involvement of lymph nodes, bone marrow, or other organs at diagnosis and for six months thereafter, confirmed by appropriate staging procedures [1]. PCLs can originate from mature T lymphocytes, B lymphocytes, or natural killer (NK) cells, and are broadly categorized as primary cutaneous T-cell lymphomas (PCTCL) and primary cutaneous B-cell lymphomas (PCBCL), representing 19% of extranodal lymphomas, with an estimated annual incidence of 1:100,000 [2,3]. PCTCLs account for about 65%, PCBCLs for 25%, and the remaining are NK cell neoplasms [4].

Cutaneous T-cell lymphomas (CTCL) represent a heterogeneous group of Non-Hodgkin lymphomas characterized by malignant T lymphocytes infiltrating the skin. Mycosis fungoides (MF) is a prominent subtype, representing primary CTCL without evidence of extra-cutaneous involvement at diagnosis [5]. The annual incidence of CTCL is estimated at 10.2 per million persons, with a higher occurrence in individuals around the fifth decade of life [6]. Clinically, MF typically manifests as red scaly patches or plaques, predominantly in sun-protected areas, progressing to thick infiltrated plaques, tumors, or leukemic disease in advanced stages [7]. Distinguishing MF from inflammatory skin conditions poses diagnostic challenges due to its variable presentation, earning it the reputation of being a dermatological chameleon [8]. Establishing an accurate diagnosis often requires multiple biopsies and consultations, underscoring the need for alternative diagnostic approaches.

In recent years, dermatologists have explored dermoscopy as a cost-effective, non-invasive tool for assessing various skin neoplasms, including inflammatory diseases and, more recently, aiding CTCL diagnosis [9,10]. Similarly, high-frequency ultrasonography (HFUS) has emerged as a non-invasive method for evaluating skin neoplasms, showing promise in diagnosing MF [11].

Differentiating primary cutaneous lymphomas from secondary cutaneous lymphomas, which arise from systemic lymphomas, is crucial due to differing therapeutic approaches, prognosis, and treatment responses [12]. Despite their relative frequency, primary cutaneous lymphomas are often overlooked by clinicians due to variability in clinical and immunohistological features [5]. Few studies and case reports describe the radiological features of these malignancies, as they are rarely evaluated with radiological investigations. 

High and ultra-high-frequency ultrasound (HFUS and UHFUS) are instrumental in the clinical management of primary cutaneous lymphomas (PCLs) by offering detailed, real-time imaging of the skin and subcutaneous layers [13,14]. 

HFUS typically penetrates to a depth of 3–4 cm, making it suitable for imaging superficial structures like skin, tendons, and superficial muscles, with a minimum effective depth of about 1 cm. On the other hand, UHFUS has a penetration depth of up to 1–2 cm, providing exceptional detail for superficial skin and small joints, with a minimum penetration depth of approximately 0.5 cm. 

The integration of UHFUS with other diagnostic methods, such as dermoscopy, allows for a comprehensive assessment of cutaneous lesions, facilitating a more precise and timely diagnosis of primary cutaneous lymphomas [15].

The application of UHFUS spans various medical fields due to its capability to deliver real-time imaging, avoid ionizing radiation exposure, and maintain consistency in lesion monitoring [16]. Recent studies advocate for the growing use of UHFUS in the diagnosis, prognosis, treatment, and monitoring of a wide range of diseases and conditions. Initial investigations by Shintani et al. [17] examined the sonographic features of healthy oral mucosa, identifying uniform patterns in the tongue and buccal mucosa. Technological advancements have since enhanced the use of high-frequency ultrasound, providing the improved detail and resolution necessary for the precise characterization of various oral mucosal lesions and structures [18,19].

Despite their relatively common occurrence, primary cutaneous lymphomas are frequently overlooked by clinicians and not included in the initial list of possible diagnoses. This is due to the significant variability in their clinical presentation and immunohistological characteristics. Since radiological assessments are seldom employed in the evaluation of these types of tumor, there is a scarcity of studies and case reports describing their imaging features in the literature. The aim of this article is to delineate the imaging characteristics of primary cutaneous lymphomas, aiding in their differentiation from cutaneous neoplasms, metastases, and benign skin lesions.

This manuscript aims to delineate the imaging characteristics of primary cutaneous lymphomas trying to depict a new classification based on their HFUS appearance.

The research protocol received approval from the local ethics committee at the University Hospital of Campania “L. Vanvitelli” and AORN “Ospedale dei Colli”, Naples, with Protocol Number 15674/i/2022. Prior to participation, all subjects provided informed consent. The study was conducted in accordance with the principles outlined in the Declaration of Helsinki for research involving human subjects.

### 1.1. Patients Criteria

Patients referred to the Oncohematological and Dermatological Unit at the University Hospital “Luigi Vanvitelli” in Naples, Italy, were considered for inclusion in this study and were enrolled between March 2022 and March 2024. Eligibility criteria included being adults aged 18 or older, having a clinical diagnosis of a skin lesion persisting for more than 15 days, being suitable for surgical biopsy, being in good health or having well-controlled chronic conditions managed with medication, and being willing to participate in the study.

Exclusion criteria were severe comorbidities resulting in a life expectancy of less than one year, pregnancy, acute or chronic conditions that would impede study participation, or refusal to provide informed consent. Additionally, reactive lymphoid hyperplasias, such as cutaneous “pseudolymphomas” and pre-lymphomatous conditions that resolved either spontaneously or after treatment with non-aggressive modalities, were excluded from the study.

### 1.2. HFUS Scan Protocol

The UHFUS scan protocol involved examining skin lesions using a 70 MHz probe, which has been available at the Institute since 2016. Each participant underwent UHFUS examination using the Vevo^®^ MD device from VisualSonics in Toronto, ON, Canada. The imaging assessment typically took about 15 to 20 min, accounting for patient preparation, application of coupling gel, and complete scanning of the affected area. The duration varied depending on the radiologist’s expertise.

Before biopsy, a highly experienced radiologist, with over 10 years of practice, conducted skin ultrasound assessments using a high-frequency probe covered with a sterile latex sheath. A small amount of ultrasound gel was applied inside the cover to maintain distance between the probe and the skin, enhancing visualization of exophytic or ulcerated lesions. Gentle pressure was applied to minimize compression distortion of the lesion’s thickness and morphology. B-mode and C-mode acquisitions were performed for each lesion using standardized presets, ensuring consistent parameters such as gain, time gain compensation, dynamic range, mechanical index, and thermal index. The scan depth and focus position were adjusted as needed to optimize scan quality.

B-mode skin images were obtained using a 48–70 MHz transducer with variable central frequencies ranging from 30 to 50 MHz. During the examination, the radiologist carefully considered potential artifacts caused by shadowing from interposed structures like air, or surface irregularities such as ulcerations. Additionally, any acoustic impedance mismatch, such as air pockets between the probe and the skin, or the presence of foreign materials like hair, could create strong echoes and obscure underlying structures.

Motion artifacts, which result from patient or transducer movement, were mitigated by instructing patients to remain as still as possible during the examination. Imaging features were retrospectively analyzed using static anonymized images stored in the institutional Picture Archival and Communication System (PACS).

For each patient, a primary skin lesion was identified based on its size or reliability in terms of echo structure and dimensions. Each indexed lesion underwent measurement of its extent at the epidermal and subepidermal levels, along with evaluation of morphology, margins, and dermal–epidermal infiltration. Vascularity was assessed using color Doppler integration for each lesion.

Initially, all patients underwent UHFUS and color Doppler examination of lesions using variable-frequency high-resolution transducers.

### 1.3. Radiological Assessment

The lesions were categorized based on their sonographic appearance into four types: “Nodules” manifest as hypoechoic nodules with a clearly defined border; “Pseudonodules” exhibit focal hypoechoic nodules with polylobulations, lacking a distinct border; “Infiltrative” lesions appear as hypoechoic, diffusely infiltrative, panniculitis-like lesions involving both the dermis and subcutaneous tissue. Two subcategories were subsequently added, allowing each of them to be further subcategorized as focal or diffuse.

### 1.4. Pathological Assessment

Histopathological assessment was performed by a pathologist with over 10 years of experience in the field. Diagnosis of primary cutaneous lymphoma was confirmed through the histopathology and immunohistochemistry (IHC) of tissue obtained via excisional biopsy with IHC conducted for B-cell markers CD19 and CD20, and T-cell markers CD3 and CD5. Macro and microscopic evaluation was performed too.

### 1.5. Data Collection

The data collection for this study received approval from the Institutional Ethics Review Board of our institution.

## 2. Results

### 2.1. Patients Caratheristics

During the study period from March 2022 to March 2024, a cohort of 88 individuals underwent examination. Their ages ranged from 23 to 67 years, with a median age of approximately 56 years, and there was a slight female predominance (male/female ratio of 3:4).

The majority (50 individuals) presented with multiple subcentimeter-sized reddish nodular lesions, while 20 individuals had larger lesions ranging from 10 to 15 mm in size. The remaining 18 patients presented with papules or skin spots. Clinical presentations varied, including plaques, papules, and nodules, often exhibiting reddish to violaceous discoloration of the overlying skin. B-cell lymphomas were commonly observed as nodules, with T-cell lymphomas manifesting as both nodules and plaques, sometimes with ulceration. Lesions ranged from solitary to multifocal, with growth rates varying from indolent to rapid, and sizes ranging from 0.4 to 6.0 cm in diameter. The lesions were frequently located on the head and neck, trunk, and upper and lower limbs.

Among the participants, fifty were identified as smokers, with an average consumption of 15.6 cigarettes per day. Twelve patients had concurrent health conditions, including twelve cases of previous breast cancer and twenty-four with liver damage related to HCV.

Seventy patients reported either no pain or mild pain (rated < 4 on the Visual Analog Scale—VAS), while fourteen patients experienced more intense pain levels (>6 on the VAS). The average VAS score for all patients was 5.1. Importantly, patients with endophytic lesions had significantly lower VAS scores (mean: 3.75 ± 3.10, *p* < 0.05) compared to those with exophytic lesions (mean: 6.33 ± 3.09). Twenty patients did not report any pain.

Ten patients experienced weight loss (4–11 kg) concurrent with a rapid increase in lesion number, size, and distribution.

Among the study participants, fifty-three patients were diagnosed with Primary T-Cell Cutaneous Lymphoma, twenty-six with Primary B-Cell Cutaneous Lymphoma, and nine with Primary NK-Cell Cutaneous Lymphoma.

### 2.2. Imaging Assessment

**Figure 1:**
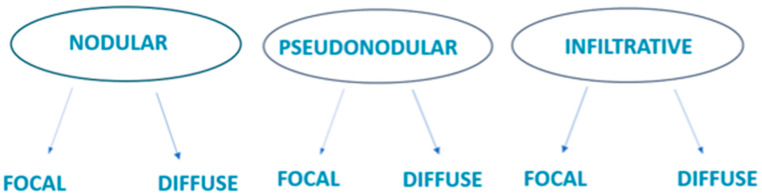
In our experiment, we developed a new classification comprising six ultrasound patterns.

Based on our observations, we categorized the lesions into distinct groups based on their ultrasound characteristics (Figure 1).

**Figure 2:**
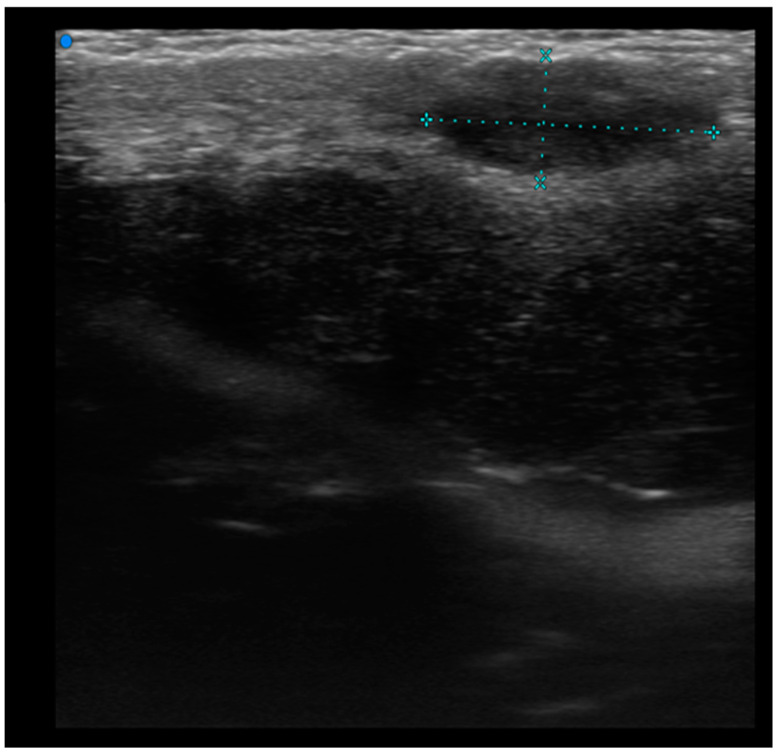
HFUS assessment of a round hypoechoic subepidermal nodular lesion showing no infiltration of deeper planes. Pathological assessment revealed it to be a B cell Primary Cutaneous Lymphoma.

**Figure 3:**
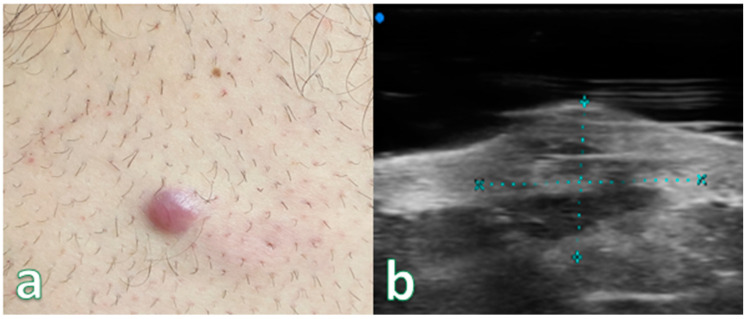
(**a**) Reddish nodular skin lesion without signs of flogisis in the anterior region of the right leg. (**b**) Examination performed with a very high-frequency ultrasound probe at 50 MHz showing iso-hypoechoic lesion with irregular and hyperechoic margins infiltrating the epidermis and the subepidermal layer highlighted with dashed blue line. Final pathologic assessment revealed to be a B-Cell Lymphoma.

**Figure 4:**
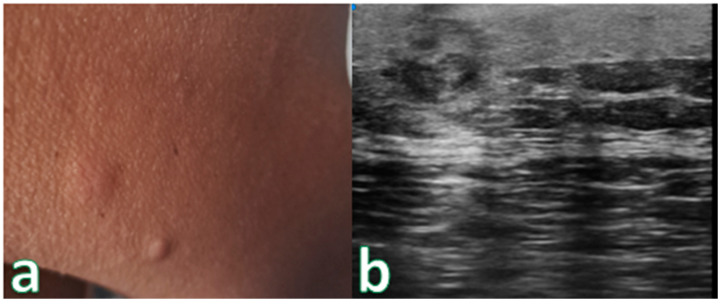
(**a**) Nodular skin lesion without signs of flogisis in the right deltoid region. (**b**) Exam performed with a very high-frequency ultrasound probe at 50 MHz: Iso-hypoechoic lesion with irregular and hyperechoic margins infiltrating the epidermis and the subepidermal layer. Pathologic Assessment revealed B-Cell Lymphoma.

Single or multiple hypoechoic nodules (Figure 2) with focal epidermal–subepidermal involvement, featuring regular margins without peripheral infiltration, were typical of B-cell lymphoma (Figure 3 and Figure 4).

**Figure 5:**
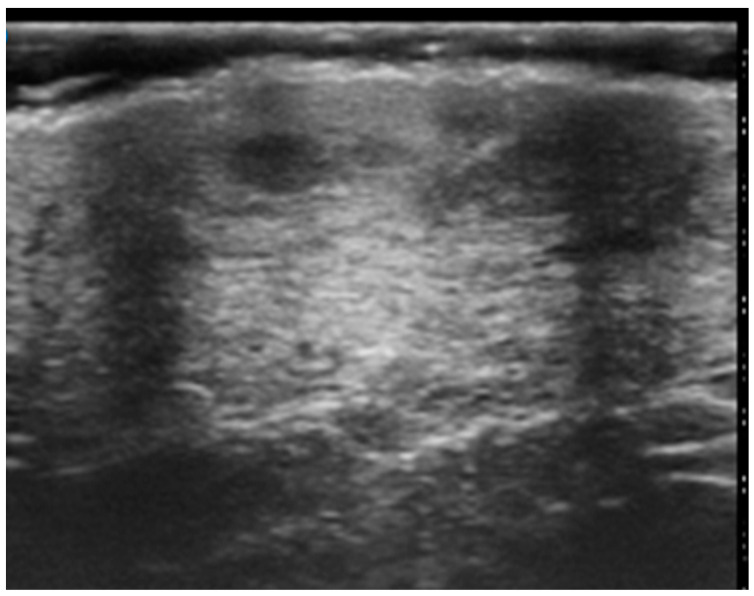
Focal pseudonodular lesion (T-cell lymphoma). Examination performed with a very high-frequency ultrasound probe at 48 MHz in the region of the right scapula: Iso-hypoechoic lesions with regular margins and epidermal localization.

Pseudonodular and nodular lesions were more frequently observed in B-cell lymphomas (70%), while diffusely infiltrative lesions were equally prevalent in both B-cell and T-cell lymphomas (50% each) (Figure 5).

**Figure 6:**
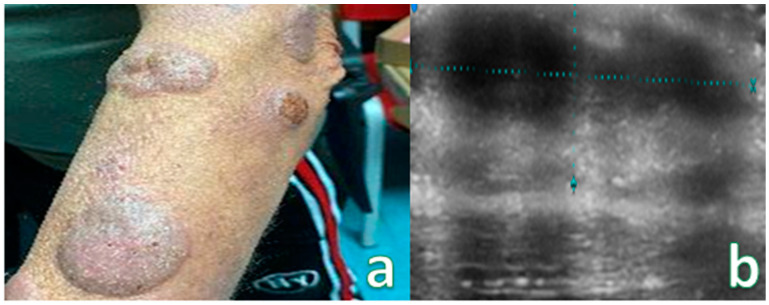
(**a**) Multiple pseudonodular lesion of the left forearm. (**b**) Examination performed with a very high-frequency ultrasound probe at 50 MHz in the anterior region of the left forearm showing iso-hypoechoic lesions with irregular margins located in the epidermal and subdermal layers highlighted by dashed blue lines. Images are low quality due to irregular surfaces of lesion.

High-Frequency ultrasound (HFUS) revealed dermal thickening in all cases, with no evidence of necrosis, calcification, or posterior acoustic shadowing in any of the lesions. Color Doppler imaging showed that initial focal infiltrative lesions were avascular, whereas nodular, pseudonodular, and diffusely infiltrative lesions were highly vascular. In contrast, nodular or multinodular lesions clustered in the epidermal and subepidermal layers with dermal involvement and clear or irregular margins, with or without peripheral infiltration, were more indicative of T-cell lymphoma. Plaque-like lesions showing iso-hypoechoic characteristics and clear margins, with or without dermal infiltration, were consistent with mycosis fungoides (Figure 6).

**Figure 7:**
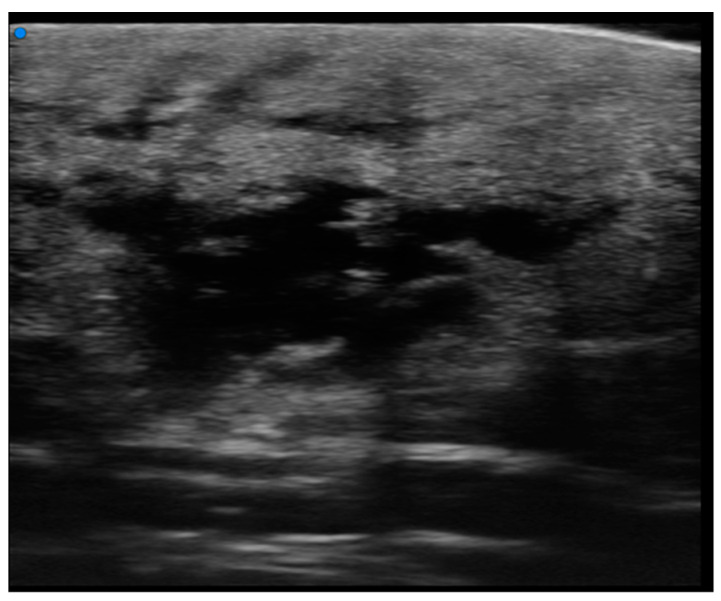
Diffuse infiltrative lesion (recurrence of B-cell lymphoma) examination performed with a very high-frequency ultrasound probe at 50 MHz in the region of the right scapula: Iso-hypoechoic lesions with nodular and multilobulated morphology and irregular margins infiltrating the epidermal and subepidermal tissue.

Cutaneous plaques were observed on HRUS as hypoechoic, diffusely infiltrative lesions (Figure 7).

**Figure 8:**
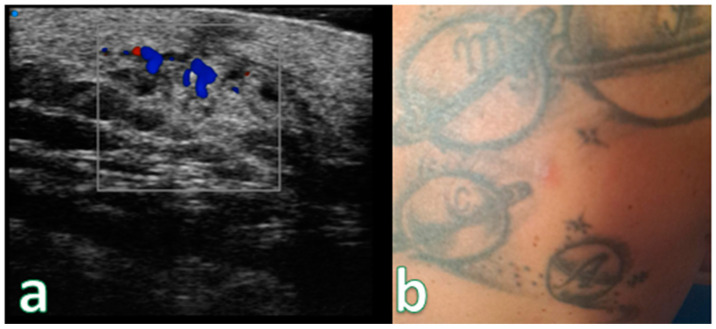
(**a**) Examination performed with a very high-frequency ultrasound probe at 48 MHz, showing iso-hypoechoic lesions with nodular and multilobulated morphology and irregular margins infiltrating the epidermal and subepidermal tissue at a single point (focal). (**b**) Reddish, indistinct, slightly exofitic nodule in the lumbar region. Color Doppler analysis showing no significative increase in the signals. Pathological assessment showed B-Cell Lymphoma (recurrence).

Only two lesions exhibited ulceration, manifested as a gap in the epidermal region with thickening of the surrounding dermis. Cutaneous papules were observed on ultra-high frequency ultrasound (UHFUS) as focal infiltrative lesions, and some of them later coalesced to form ill-defined pseudonodules (Figure 8).

Hence, we categorized the lesions as infiltrative focal or nodular lesions, non-infiltrative focal lesions, infiltrative pseudonodular lesions, non-infiltrative pseudonodular lesions, and diffusely infiltrative lesions. Lesions displaying dermal infiltration and irregular margins demonstrated an increase in vascular signal on Doppler integration. Notably, none of the lesions showed calcifications in our study.

## 3. Discussion

The term “primary cutaneous lymphoma” refers to non-Hodgkin’s lymphomas (NHLs) affecting the skin without evidence of involvement beyond the skin at the time of diagnosis and initial staging evaluation [20]. The skin ranks as the second most common site affected in primary extranodal NHL, while cutaneous involvement is rare in Hodgkin’s disease [21]. A notable distinction exists between primary and secondary cutaneous lymphomas, with primary cutaneous lymphomas typically displaying an indolent course and better prognosis compared to secondary cutaneous lymphomas [22]. Primary cutaneous lymphomas typically affect middle-aged to elderly individuals, with a slight male predominance [23]. Recent studies indicate an increase in the relative frequency of primary cutaneous B cell lymphomas (PCBCL), particularly in older populations compared to primary cutaneous T cell lymphomas (PCTCL) [24]. Presentation varies, with lesions appearing as red to violaceous patches, papules, and skin nodules, generally without scaling or ulceration, except in cases of T cell lymphomas, which may present with ulceration or cellulitis-like swelling [8]. Lesions can be solitary or multiple, with multiple lesions more common in PCTCL than in PCBCL. Common locations include the trunk, legs, arms, and head and neck regions [7].

Neoplastic cells in primary cutaneous lymphomas express specific clusters of designation (CD) markers, essential for diagnosis and classification, making immunohistochemistry (IHC) crucial to their evaluation [25].

In addition to primary CBCL, the skin can serve as the initial site of presentation for low-grade systemic BCLs. Previous studies have defined CBCL outcomes by the absence of systemic disease evidence within six months of the initial presentation [26]. This definition excludes cases where skin involvement is a manifestation of systemic BCLs and those with rapid disease progression [27].

The true frequency of skin being the initial manifestation of systemic BCLs is unknown, although it is believed to be uncommon. The latest guidelines from the National Comprehensive Cancer Network recommend a thorough evaluation for individuals with a cutaneous presentation of indolent BCLs [16]. The diagnosis of CTCL often experiences a delay of up to several years. Currently, diagnosis relies on the clinical evaluation and histopathologic analysis conducted by experienced dermatologists and pathologists [28]. Dermoscopy and HFUS are both non-invasive diagnostic tools. Dermatologists commonly use hand-held dermatoscopes, which are affordable and accessible at all levels of training. However, the use of HFUS for assessing cutaneous lesions is not widely integrated into routine dermatologic examinations [29]. Our study highlights the practicality of HFUS in a clinical setting and its ability to distinguish between CTCL lesions and inflammatory skin conditions. 

Reflectance Confocal Microscopy (RCM) and Line-field Optical Coherence Tomography (LC-OCT) offer superior cellular resolution but have limited penetration compared to HFUS. While RCM and LC-OCT are ideal for imaging superficial cellular details, HFUS is more versatile for visualizing slightly deeper structures. Ulcerations can cause artifacts across all modalities, but each technique has its strengths, depending on the required depth and resolution.

Because of its diverse range of symptoms, distinguishing Mycosis Fungoides from inflammatory skin diseases can be difficult. Therefore, Mycosis Fungoides has been referred to as a significant mimic in dermatology [1]. Experienced clinicians and a comprehensive approach combining clinical evaluation, histopathology, and molecular biology are necessary to accurately diagnose the condition [2]. Recent data obtained through prospective studies have revealed that there is an average delay of three years between the onset of initial symptoms and the final diagnosis of Micosis fungoide [24].

In our current study, we used an ultra-high frequency transducer with a maximum frequency of 70 Mhz. In our experiment, we examined and described the lesions as single or multiple hypoechoic nodular lesions with focal epidermal–subepidermal location and regular margins without peripheral infiltration, which are typical of B-cell lymphoma. Nodular or multinodular cluster lesions with epidermal–subepidermal location and involvement of the dermis with clear or irregular margins, with or without peripheral infiltration, are more evident in T-cell lymphoma. Plaque lesions with iso-hypoechoic characteristics and clear margins, with or without dermal infiltration, are typical of mycosis fungoides. Among them, only one lesion showed ulceration, identified as a gap in the epidermal region and thickening of the surrounding dermis. 

Therefore, we described them and proposed the following classification of cutaneous lymphomatous lesions as focal or diffuse nodular, focal or diffuse pseudonodular, focal or diffuse infiltrative. This integrates the semiotics of the marginal region and the infiltration of the cutaneous and epidermal layers. Lesions with dermal infiltration and irregular margins showed increased vascular signal on Doppler integration. None of our lesions presented calcifications. Although high-resolution ultrasound (HRUS) with color Doppler using high-frequency transducers is increasingly utilized in imaging cutaneous lesions, data on imaging primary cutaneous lymphomas remain limited. Early studies describe B cell lymphomas producing sharply demarcated infiltrates in a “focal nodular” pattern, while T cell lymphomas cause diffuse dermal involvement. However, these patterns are not mutually exclusive, and sonographic features do not always correlate with histological diagnosis. Common findings include dermal thickening, absence of calcifications, and central necrosis. Lesions initially appear as hypoechoic infiltrations, becoming vascular as they progress. Primary cutaneous lymphomas often present as multifocal lesions, with physical examination sometimes underestimating disease burden. While HRUS can delineate sonographic features and lesion extent within cutaneous layers, it does not show disease progression beyond superficial lesions and does not confirm diagnosis definitively. Accurate staging and disease definition, particularly regarding systemic dissemination, are crucial for prognosis and treatment planning. Cross-sectional imaging modalities like CT and MRI, while limited by the small and superficial nature of cutaneous lesions, may aid in assessing lesion size, regional multifocality, and local staging. Positron Emission Tomography–Computed Tomography (PET–CT), which combines metabolic information from FDG PET with anatomical details from CT, is superior to CT alone in staging, restaging, and detecting extranodal involvement in lymphomas. PET–CT imaging frequently reveals increased FDG uptake in cutaneous lesions, aiding in staging, post-therapeutic monitoring, and detecting extracutaneous involvement. In our series, one case of PCBCL revealed extensive skeletal metastases on PET–CT, a rare finding not commonly reported in the literature. 

The International Society for Cutaneous Lymphomas and the European Organization for Research and Treatment of Cancer have developed a TNM (tumor, node, metastasis) system to document the anatomical extent of cutaneous lymphomas, providing guidelines for appropriate management [1]. Additionally, the International Prognostic Index, considering factors such as age, serum LDH levels, extent of skin involvement, and other markers, aids in predicting prognosis across all non-Hodgkin lymphoma subtypes. Presently, CT, whole-body PET (18F-FDG), and ultrasound of the neck are included in the evaluation guidelines for primary cutaneous lymphomas (PCL), while high-resolution ultrasound (HRUS) of primary lesions plays no role in diagnostic assessment or prognostic index determination [30]. With advancements in transducer technology and expanding indications for ultrasonography in dermatological lesion assessment, we anticipate increased utilization of HRUS in evaluating primary lesions and monitoring the progression of cutaneous lymphomas. Traditionally, the diagnosis of numerous skin diseases, whether they present focal or diffuse characteristics, has heavily relied upon physical examination findings [31]. However, research studies have highlighted that High-Frequency US (HFUS) surpasses clinical examination alone by furnishing invaluable insights into the detection and precise measurement of both clinical and subclinical cutaneous lesions [29].

## 4. Conclusions

High Frequency Ultrasound (HFUS) represents a pioneering approach in the study and identification of cutaneous lymphomas. Given the rarity of the disease and the limited radiological literature, our case series is currently distinctive. Drawing on our extensive expertise in this field, we have developed standardized parameters that effectively catalog and identify typical lesions associated with these conditions. While dedication and collaboration with specialized teams are essential, our classification could potentially integrate radiologists into post-therapeutic decision-making processes.

The use of HFUS significantly enhances the diagnostic process for suspected cases of primary cutaneous lymphoma. HFUS offers several advantages in diagnosing these lymphomas. Firstly, it allows for a non-invasive examination of the skin, providing a more comfortable experience for patients. Additionally, HFUS provides high-resolution imaging of the skin layers, enabling detailed analysis of affected areas. This precision assists in distinguishing between benign and malignant lesions, thereby facilitating accurate diagnosis of primary cutaneous lymphomas. Moreover, HFUS can monitor the progression of cutaneous lymphomas over time. Repeated HFUS examinations track changes in lesions, assess treatment effectiveness, and guide patient care decisions.

In conclusion, HFUS represents a crucial advancement in diagnosing cutaneous lymphomas. Its non-invasive nature, high-resolution imaging capabilities, and ability to monitor disease progression make it invaluable in diagnosing suspected cases of primary cutaneous lymphoma. Our experiment and standardized parameters ensure the accurate identification and cataloging of typical lesions associated with this condition.

## Data Availability

Data available on demand to the corresponding author.
